# Precision Nephrology in Patients with Diabetes and Chronic Kidney Disease

**DOI:** 10.3390/ijms23105719

**Published:** 2022-05-20

**Authors:** Michele Provenzano, Federica Maritati, Chiara Abenavoli, Claudia Bini, Valeria Corradetti, Gaetano La Manna, Giorgia Comai

**Affiliations:** Nephrology, Dialysis and Renal Transplant Unit, IRCCS—Azienda Ospedaliero-Universitaria di Bologna, Alma Mater Studiorum University of Bologna, 40138 Bologna, Italy; federica.maritati@aosp.bo.it (F.M.); chiara.abenavoli@studio.unibo.it (C.A.); claudia.bini@aosp.bo.it (C.B.); valeria.corradetti@gmail.com (V.C.); giorgia.comai@aosp.bo.it (G.C.)

**Keywords:** personalized medicine, end stage kidney disease, cardiovascular risk, proteinuria, eGFR

## Abstract

Diabetes is the leading cause of kidney failure and specifically, diabetic kidney disease (DKD) occurs in up to 30% of all diabetic patients. Kidney disease attributed to diabetes is a major contributor to the global burden of the disease in terms of clinical and socio-economic impact, not only because of the risk of progression to End-Stage Kidney Disease (ESKD), but also because of the associated increase in cardiovascular (CV) risk. Despite the introduction of novel treatments that allow us to reduce the risk of future outcomes, a striking residual cardiorenal risk has been reported. This risk is explained by both the heterogeneity of DKD and the individual variability in response to nephroprotective treatments. Strategies that have been proposed to improve DKD patient care are to develop novel biomarkers that classify with greater accuracy patients with respect to their future risk (prognostic) and biomarkers that are able to predict the response to nephroprotective treatment (predictive). In this review, we summarize the principal prognostic biomarkers of type 1 and type 2 diabetes and the novel markers that help clinicians to individualize treatments and the basis of the characteristics that predict an optimal response.

## 1. Introduction

Diabetes is a major cause of Chronic Kidney Disease (CKD) and the leading cause of End-Stage Kidney Disease (ESKD) [1,2,3]. Overall, about 50% of patients with type 2 diabetes and about one-third of those with type 1 diabetes will develop CKD over time [4,5]. Chronic Kidney Disease in patients with diabetes (also called diabetic kidney disease, DKD) is defined following the Kidney Disease Improving Global Outcomes Work Group (KDIGO) guidelines, as the presence of either decreased kidney function (estimated glomerular filtration rate (eGFR) < 60 mL/min/1.73 m^2^) and/or albuminuria, which is considered the main marker of kidney damage [6]. This classification encompasses patients who do not rigorously follow the classical stages of DKD, namely progression from normal to increased albuminuria and, then to a low eGFR. In fact, in large epidemiologic studies, many diabetic patients have a significant reduction in eGFR without albuminuria, or vice-versa, raised albuminuria levels but no decrease in eGFR [7]. Etiology of CKD in patients with diabetes is complex and includes multiple mechanisms such as glomerular hemodynamics (i.e., glomerular hyperfiltration), inflammation, oxidative stress and fibrosis [8,9,10]. Regardless of the mechanism of damage, it has been shown that DKD patients represent a clinical subgroup with an extremely poor prognosis [11]. A large meta-analysis of the Chronic Kidney Disease Prognosis Consortium, including more than 1 million patients, compared patients with and without diabetes across the same cut-offs of eGFR and albuminuria, and found that, keeping patients without diabetes as a reference, those with diabetes were at an increased, up to 2-fold, risk for all-cause and cardiovascular (CV) mortality [12]. Moving from these dramatic evidences, a number of clinical trials have been carried out with the aim of reducing CV risk, mortality and slowing progression to ESKD, in DKD patients [13,14,15,16,17,18,19]. All these trials have answered the question of whether DKD patients may benefit from the addition of nephroprotective drugs (e.g., blood pressure lowering drugs, albuminuria lowering drugs, drugs targeting hemoglobin levels, antioxidant inflammation modulators) to the standard-of-care represented by the renin-angiotensin-system inhibitors (RAASi). The SONAR, the CREDENCE and the FIDELIO-DKD trials are particularly relevant since they demonstrated that sodium-glucose co-transporter inhibitors (SGLT2is), endothelin-1 receptor antagonists (ERA) and the novel non-steroidal mineralocorticoid receptor antagonist (MRA) confer a reduction of risk of progression to ESKD in DKD patients already treated with RAASi. However, going deeper into these studies, it should be noted that the residual risk of future events remains high. In the SONAR study, the cardiorenal event rate was 5.2% patients/year in the atrasentan arm and 6.1% patients/year in the placebo arm [17]. Although this difference was statistically significant in favor of atrasentan, a not trivial number of patients were still at risk, even if they had been treated more. The modern suggestion is that we are relenting CKD progression rather than treating the underlying kidney damage. A further effort is, thus, required. One fascinating strategy consists of individualizing prognosis and treatment in DKD patients—so-called precision (or personalized) medicine [20]. This consists of the use of biomarkers or clinical measures that can help a clinician make the true decision in terms of treatment to prescribe, based on the likelihood of each patient to respond to that drug, and also to plan the follow-up in the clinic based on the true estimation of future prognosis given the patient’s characteristics [21,22,23,24]. Previous studies have examined this topic and interesting findings have been reported around diagnostic and prognostic aspects of personalized medicine in DKD patients [23,24]. We here present a narrative review, which summarizes the main evidence around precision medicine in the context of DKD, with regards to prognosis and prediction.

## 2. Diabetic Kidney Disease: Definition and Prognosis

**Figure 1:**
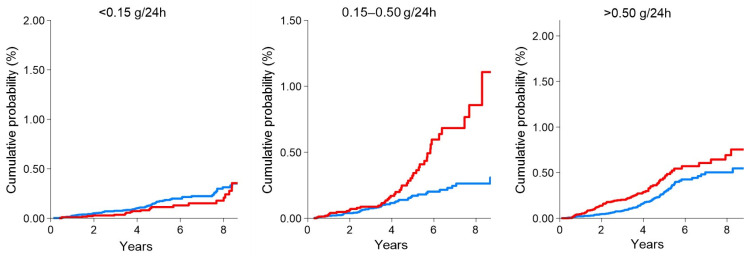
Cumulative probability of fatal and non-fatal cardiovascular (CV) events in DKD patients (red line) compared with non-DKD patients (blue line) by 24 h proteinuria categories. The presence of diabetes significantly increased the risk of CV events even for mild proteinuria values (0.15–0.50 g/24 h). This figure was derived from the individual data of a cohort of CKD patients followed by Nephrologists in Italy. Data from the Italian Multicohort of Chronic Kidney Disease patients followed by nephrologists (Minutolo et al.) [11]. The curves were built using the Nelson Aalen estimator of the cumulative event probability over time. *p*-values were: 0.208 (proteinuria < 0.15 g/24 h); <0.001 (proteinuria 0.15–0.50 g/24 h); <0.001 (proteinuria > 0.50 g/24 h).

Diabetes Kidney Disease is a heterogeneous disease and refers to patients with the concomitant presence of diabetes and CKD [7]. It is seldom possible, in clinical practice and epidemiology, to discern between ‘diabetic nephropathy’ and the presence of kidney damage in the context of diabetes, so the diction DKD encompasses both these conditions. Such a definition is helpful, since most cases of DKD do not rigorously follow the Mogensen’s phases of the disease, depicted in 1980, and thus notwithstanding, they remain at increased CV and renal risk. What is known from the United Kingdom Prospective Diabetes Study (UKPDS), a large longitudinal cohort study following diabetic patients over time, is that about one third of these patients develop kidney damage, detected with the presence of albuminuria of eGFR reduction [25]. In patients referred to nephrologists, the prevalence of DKD ranged between 14–30% [26,27]. These data are alarming if it is considered that the global prevalence of diabetes has more than doubled in men and has increased by 60% in women [28]. Among the causes of ESKD in the United States, DKD was confirmed to lead the ranking, with more than 150 cases per million persons/year [29]. In addition to the high-risk for CKD progression to EKSD, patients with DKD have a very scarce CV prognosis. According to previous data derived from very large cohorts, patients with DKD had a 2.7 higher risk of myocardial infarction during follow-up in the Alberta Kidney Disease Network (AKDN) cohort and National Health and Nutrition Examination Survey (NHANES) population [30]. This risk was strikingly higher than that observed in patients with only diabetes (2.0) or only CKD (1.4) in the same cohorts. Similar estimates were reported for all-cause mortality. Cardiovascular events in DKD patients can be predicted with traditional and non-traditional risk factors. Among the first, a remarkable role is played by age, blood pressure, serum lipids levels, and smoking habit [31]. However, recent studies have interestingly highlighted that an equally important role should be given to non-traditional risk factors such as proteinuria (or albuminuria) and eGFR [32]. Proteinuria and eGFR are two kidney measures which enter in the principal classification of CKD, according to the current guidelines, and have a major impact on therapeutic decisions [6]. Based on eGFR levels (mL/min/1.73 m^2^), CKD is classified into six risk categories: G1 (≥90), G2 (60–89), G3a (45–59), G3b (30–44), G4 (15–29) and G5 (<15). Overall, stages G1–G3a configure mild–moderate CKD; stages G3b–G4 moderate–severe disease; and G5 refers to kidney failure, namely, the most advanced stage of CKD. Each eGFR-based category (G) is divided into three sub-categories (A1, A2, and A3) according to the degree of albuminuria (or proteinuria). Albuminuria can be measured in clinical practice through the albumin-to-creatinine ratio (ACR, expressed as mg/g) or via the 24 h urine collection (in this case the output will be reported as mg/24 h) [33]. Proteinuria measurement is similar with the use of the protein-to-creatinine ratio (PCR, mg/g) or 24 h proteinuria excretion (mg/24 h). A1 (normal–mild) refers to albuminuria levels of <30 mg/g (or mg/24 h), A2 (moderate, or micro-albuminuria) to 30–300 mg/g (or mg/24 h) and A3 (severe, or macro-albuminuria) to values greater than 300 mg/g (or mg/24 h). These three categories can be created using 24 h proteinuria by replacing the threshold with <150 mg/24 h (normal-mild), 150–500 mg/24 h (moderate) and >500 mg/24 h (severe) [6]. The final result of such a stratification is a combination of G and A categories (GA stages) which detect categories of patients with different prognosis. In particular, risk of CKD progression and CV events increase as albuminuria increases (moving from A1 to A3) and as eGFR falls down (from G1 to G5) [32]. As a consequence of such an interaction between albuminuria and eGFR, the highest event rates are present in patients with worse G and A categories at the same time. However, this classification also showed that albuminuria and eGFR should be both carefully monitored, since, for example, patients with a normal–mild reduction of kidney function (G grade) have a non-negligible future risk if albuminuria is severe (A3) [34]. In patients with diabetes, proteinuria acts as a modulator of future CV risk. In fact, in a cohort of CKD patients followed by nephrologists, risk for CV events (myocardial infarction, stroke, peripheral vascular disease and heart failure) started for mild–moderate 24 h-proteinuria (0.150–0.500 g/24 h) in DKD patients if compared with CKD patients without diabetes where the risk started from the severe proteinuria category (>0.500 g/24 h) (Figure 1) [11].

## 3. Complexities Underlying Diabetic Kidney Disease: Molecular Mechanisms of Damage

The pathophysiology of DKD is widely complex and heterogeneous. It is well demonstrated that the altered metabolic milieu is responsible for the initial damage that in turn leads to DKD [35]. The presence of extracellular hyperglycemia determines an increase of the concentration of intracellular glucose. In diabetic subjects, intracellular glucose is preferentially oxidized via the fructose 6-phosphate, hexosamine and via the polyol pathways [36]. The change in type of intracellular glucose metabolism together with the activation of non-enzymatic pathways generate a spectrum of aberrant substrates, namely advanced glycation end-products (AGE) and reactive oxygen species (ROS). These substrates alter the gene expression over time and trigger a phenomenon known as ‘epigenetic reprogramming’ which consists, for these patients, in the perpetual and long-term activation of pro-fibrotic genes [37]. Among them, the hyperactivation of the chromatin-modifying enzyme PARP1 has been found in DKD patients [35]. Moreover, the aforementioned diabetic milieu is characterized by an increase in circulating levels of vasodilators such as nitric oxide and prostaglandins and a concomitant increase in vasoconstrictors like angiotensin II and endothelin 1. This imbalance ultimately results in the vasoconstrictions of efferent glomerular arterioles and, in consequence, in the abnormally elevated GFR that marks the initial phase of DKD. In addition to the hemodynamic effect, the imbalance of molecular paths also damage the structure and function of the glomerular capillary wall. It has been reported that the increased endothelin 1 and vascular endothelial growth factor (VEGF) levels, stimulated by the hyper-activation of Protein Kinase C (PKC), promote the disruption of glomerular endothelial cells and glycocalyx, with altered selectivity and development of proteinuria, which exerts per se toxic effects on the tubule kidney cells [33,38]. The release of ROS and cytokines, like transforming growth factor-β1 (TGF-β1), platelet-derived growth factor (PDGF) and Tumor necrosis factor-α (TNF-α) promote the apoptosis of podocytes, a crucial step in the pathogenesis of DKD since these cells, which are instrumental in the control of a glomerular filtration process, are terminally differentiated cells and thus they cannot be directly replaced [39]. It has been demonstrated that the surviving podocytes experiment with a cytoskeleton reorganization, and increase dimension and spread to cover the glomerular-basal membrane. This occurs to compensate for the function of podocytes lost. The molecular mechanisms underlying these changes have been reported. The podocyte hypertrophy is mediated by mTOR kinase, which acts through the substrates S6 kinase1 and 4E-binding protein 1 [40]. The AMP-activated protein kinase (AMPK), Phosphatase and tensin homolog (PTEN) and Akt2 signaling regulate mTOR activation and represent potential targets of novel drugs for reducing glomerular damage in DKD patients. Several other molecular mechanisms related to inflammation and oxidative stress in DKD have been demonstrated. A relevant amount of inflammatory stimuli in DKD derives from the macrophage infiltration in the kidney, whose degree is directly correlated with a poor kidney prognosis [41]. The macrophages are able to release several cytokines such as TGF-β1 and TNF-α. The TGF-β1 released by macrophages, together with that synthesized by mesangial cells upon hyperglycemic/angiotensin-II stimuli, promotes mesangial cell hypertrophy and matrix accumulation across both kidney glomeruli and tubules with a mechanism at least in part mediated by connective tissue growth factor (CTGF) [42]. The interest in the activation of macrophage in DKD was confirmed by a clinical study in patients with DKD and albuminuria who were randomized to NOX-E36, a monocyte-chemotactic protein 1 (MCP-1) inhibitor [43]. MCP-1 is one of the key chemokines which regulates the migration and activation of macrophages. Albeit that the difference in albuminuria reduction between intervention and placebo was overall not significant, NOX-E36 was demonstrated to decrease urine albumin excretion (ACR) by 29% in the first three months of treatment, thus stimulating future research in this direction. One of the points deserving further reflection is that, in DKD, not all the described mechanisms of damage are active in the same patients and at the same stage of disease [22]. Even more importantly, it has been hypothesized that the pathophysiology of DKD varies during time within the same individual [44]. Several methods, such as biological vocabularies, molecular pathways and molecular networks, can be used to simplify the large amount of information derived from ‘omics’ techniques [22]. Irrespective of the technique used, it would be extremely important to develop novel biomarkers that reveal the mechanism of damage active in a specific DKD patient, since this may guide risk stratification and treatment.

## 4. Old and Novel Treatments Available for Reducing Risk in Patients with Diabetes and CKD

The first clinical trials in patients with DKD date back to early 2000 [45]. Three main studies, i.e., the Collaborative Study Group (CSG) Captopril trial, the IDNT and the RENAAL trials, have shown the efficacy and safety of angiotensin-converting-enzyme inhibitors (ACEi) and Angiotensin Receptor Blockers (ARBs) in slowing CKD progression in these patients [13,14,46]. On average, ACEi and ARBs conferred around 20% risk reduction of ESKD as compared with standard-of-care. After these important discoveries, the use of RAASi in clinical practice has shown a rapid diffusion. However, post-hoc analyses of these trials have subsequently reported that up to 40% of patients do not respond to ACEi or ARBs in terms of albuminuria reduction [47,48]. This variability in response (true variability, also called non-random variation) is independent of the day-to-day fluctuations (random variation) in albuminuria and is partially explainable by many factors, such as adherence to treatment, and albuminuria levels at the moment of start-of-treatment with RAASi [33]. For this reason, several trials have been carried out in the past two decades with the important aim of reducing this residual cardiorenal risk in DKD patients as strongly as possible. Two positive large trials, specifically conducted in DKD patients, were published in 2019 (after two decades) [17,18]. These trials have strongly demonstrated that SGLT2 inhibitors and selective ERA have a significant role in relenting CKD progression. SGLT2 inhibitors are antagonists of the SGLT-2 co-transporter located in the early proximal renal tubule, which is responsible for most (90%) of the reabsorption of filtered glucose. As is already known, in diabetes, there is an increase in expression of SGLTs, along with the growth of proximal tubules, as a consequence of hyperglycemia and enhanced intrarenal synthesis of Angiotensin II. The enhanced expression of SGLT proteins and mRNA, also demonstrated in tubular cells, contributes to the increased glucose reabsorption observed in diabetic patients. SGLT2 inhibitors determine an increase of urine excretion of glucose and thus an improvement of glycemic control, but they also cause a restoration of the normal tubule–glomerular feedback (TGF), a mechanism potentially associated with the long-term protection on the kidney. Furthermore, SGLT2 inhibitors have anti-inflammatory and anti-fibrotic effects, leading to a reduction in the amount of ROS, glomerulosclerosis and tubule-interstitial fibrosis. Recently, it has been well demonstrated that the SGLT2i dapagliflozin reduced the renal resistive index, with a potential improvement of endothelial function in the kidney [49,50]. The CREDENCE trial and the Canagliflozin Cardiovascular Assessment Study (CANVAS) trial demonstrated that the SGLT2i canagliflozin confers a 30% lower risk of renal events and a 15% risk reduction of fatal and non-fatal CV events when added to the standard of care (one ACE or ARB) [18,51]. Another drug class that raised interest in treating DKD is represented by the ERA. ERA are selective antagonists of endothelin-1 receptor A, whose activation has been associated with the development of albuminuria and glomerulosclerosis in conditions of increased production of endothelin-1, as it occurs in CKD patients. Moreover, endothelin-1 binding to ET receptors type A (ETAr) promotes oxidative stress, vasoconstriction, inflammation, cell proliferation, podocyte activation and stimulation of angiotensin II, all mechanisms that worsen the progression of renal damage over time [52]. Despite the negative results reached with avosentan, the more selective atrasentan has demonstrated, in the SONAR trial, to confer a further 35% risk reduction of renal events (i.e., doubling of serum creatinine or ESKD) in addition to RAASi use [17,53]. Unfortunately, atrasentan was also associated with an increase in fluid retention and hypervolemia as depicted by the increase in Brain-Natriuretic-Peptide (BNP) in the atrasentan versus placebo group. More recently, very positive results have emerged from the FIDELIO-DKD trial [19]. In this trial, about 5700 patients suffering from DKD were randomized to receive the non-steroidal mineralocorticoid receptor antagonist (MRA) finerenone or the standard-of-care (RAASi) and found that the finerenone group had a 20% lower risk of developing renal events. The non-steroidal MRA, like the steroidal MRA, such as Spironolactone and Eplerenone, contrasts aldosterone binding to its receptors and thus leads to a degradation of ENaC channels with consequent natriuresis. The advantage of this novel class is the greater selectivity and affinity for the mineralocorticoid receptor and a lower rate of serious adverse events, such as hyperkalemia, gynecomastia and worsening kidney function. All these studies answer the question of whether the addition of novel drug classes to patients with a residual risk of CKD progression may confer nephroprotection as compared to the standard treatments. However, none of these studies have shown thus far whether the combination of ERA, MRA and the novel SGLT2is may further decrease risk and in what patients these combinations work. This hypothesis is more than intriguing when considering that these drugs share the main mechanism of decreasing urine albumin excretion, while reducing or abolishing their adverse events each other. In fact, the natriuretic effect of SLGT2 may contrast the fluid retention mediated by the ERA via the endothelin receptor B and the hyperkalemic effect mediated by the MRAs. Studies testing these fascinating hypotheses are eagerly expected in the near future. Testing the additive effect of multiple treatments perfectly fits with the precision medicine aim. Positive results in DKD patients were also reached with the use of Glucagon-like peptide-1 receptor agonists (GLP1-RA) [54]. These agents stimulate the incretin GLP1 receptors and thus stimulate insulin secretion from pancreatic β-cells and reduce glucagon release [55]. The AWARD-7 trial was the first randomized study evaluating the efficacy and safety of the GLP1-RA dulaglutide in patients with DKD [56]. This study showed that treatment with dulaglutide, at both doses of 0.75 mg and 1.5 mg per day, was associated with a slower eGFR decline as compared to insulin glargine. Next, the AMPLITUDE-O clinical trial was carried out with the aim of comparing major CV and kidney (decrease of eGFR or increase in albuminuria) outcomes in diabetic patients with either a previous history of CV disease or with the current presence of CKD randomized to the GLP1-RA efpeglenatide or placebo [57]. A risk reduction of about 30% was reported in the efpeglenatide group, for the onset of both CV and kidney events. The aforementioned studies reported a CV and kidney protection associated with the start of treatments with different mechanisms of action. However, the patients who particularly benefit from these treatments are those who manifest a positive response in the first months of treatment, in term of albuminuria reduction, HbA1c reduction and/or blood pressure reduction. A post-hoc analysis of the RENAAL trial showed that, in the losartan arm, the magnitude of nephroprotection (ESKD risk reduction) was directly proportional to the amount of albuminuria reduction early after treatment initiation [58]. Even more impressive, the same findings were reported from the ALTITUDE database, albeit that this was a ‘negative’ study. In this study, patients treated with aliskiren + ACE/ARB had less than half the risk of CKD progression as compared with those treated with ACE/ARB alone [59]. What we learned from these relevant studies is that within each treatment group, there was a consistent variability in progression. The challenge for future studies is to reduce and minimize variability in response to the well-known biomarkers. Intriguingly, it has been well demonstrated that a non-negligible proportion of DKD patients progress to the more advanced stages of CKD (3 to 5) despite the ACE/ARB-induced albuminuria reduction [60,61]. This opposite and controversial scenario reveals that DKD is a multifactorial and multi-marker-based disease, and thus, that finding novel prognostic and predictive biomarkers is crucial.

## 5. Biomarkers and New Tools to Improve Individual Risk Prediction in Patients with Diabetes and CKD

The KDIGO guidelines classify DKD (and CKD as well) on the basis of albuminuria and eGFR categories. This approach has been shown to be clinical useful, especially in detecting patients with DKD who deserve to be referred to a nephrologist in large General Population cohorts [6,12]. At the same time, it was criticized for being considered as a “reductionist” method on behalf of many nephrologists. In fact, the albuminuria-eGFR classification does not encompass all the mechanisms and risk factors that are active in DKD patients [62]. In the context of prognosis, the final aim of precision medicine is the possibility to find and characterize subgroups of patients with the same disease but different risks of future outcomes. Type 1 diabetes is classically defined as an immune-mediated destruction of pancreatic β-cells, which leads to a discontinuation of insulin production [63]. Hence, this is usually seen as the trigger of a complete loss of blood glucose level regulation with the needs of exogenic insulin substitution treatment. However, biomarker analysis revealed that patients’ categories with different future risk can be detected. The inactive circulating peptides mid-regional proANP (MR-proANP) and N-terminal proBNP (NT-proBNP) have been found to be associated with about a 2-fold increased risk of EKSD, CV events and all-cause mortality in patients with type 1 diabetes, regardless of the main traditional risk factors such as age, gender and eGFR levels. Importantly, this association was confirmed in type 1 diabetes patients who were followed for many (>6) years [64]. The principal hypotheses underlying the strict association between MR-proANP and NT-proBNP with CV and renal risk have been related to the evidence that these peptides are released in response to stressful stimuli, such as volume overload or stretching of cardiac cells. Another class of biomarkers of growing interest is represented by the cardiac troponins, proteins which play a pivotal role in the muscular contraction and which are released in blood circulation in response to myocyte injury or necrosis. Among them, blood levels of the high-sensitivity cardiac troponin-T (hs-cTnT) were significant predictive (with about 40% more risk for each unit increase) on CV events over time, in patients with type 1 diabetes [65]. Intriguingly, the strength of association between hs-cTnT and NT-proBNP and CV risk is modified by gender, being hs-cTnT more strongly predictive in men and NT-proBNP in women [65]. Beyond the reason underlying this pattern, which is still unclear, such a finding is a good example of how many factors should be considered together to personalize prognosis of DKD patients, keeping in mind that differences are present with respect to such a fundamental characteristic as gender. Copeptin is a peptide which derives from the same precursor of arginine vasopressin and is considered a useful biomarker of several pathologic conditions such as myocardial infarction, atherosclerosis and ischemic stroke [66]. Recent observational studies have discovered that higher blood levels of copeptin are strictly associated with the development of atherosclerosis, arterial stiffness and kidney damage in patients with type 1 diabetes [67,68]. In particular, patients with the highest levels of copeptin have concomitantly increased levels of albuminuria, the main marker of kidney damage [69]. Moreover, a prospective analysis of the Steno Diabetes Center also showed that copeptin predicts CKD progression (ESKD and 30% eGFR decline) in a huge population of more than 600 type 1 diabetic patients [70]. A protein similar to albumin, namely the urinary angiotensinogen (AGT), has shown very interesting and promising results as early markers of disease severity in type 1 diabetes [71]. Urinary AGT level increases in the presence of kidney damage, because of filtration and intra-renal formation. Studies in type 1 diabetes have demonstrated that urinary levels of ATG predict eGFR decline and ESKD, regardless of baseline levels of albuminuria [72].

Genomics contributed to improve prognostic estimates in patients with type 1 diabetes. One interesting approach derives from the genome-wide association studies (GWAS) analysis, a tool that allows us to evaluate the association between a combination of single nucleotide polymorphisms and a specific disease status or outcome. A large GWAS analysis involving more than 19,000 patients detected 16 loci associated with CKD progression. Among them, the SNP variant rs55703767, responsible for a mutation in the collagen type IV alpha 3 chain (*COL4A3*), was the variant with the strongest association with kidney damage and CKD progression [73]. Type 1 diabetic patients with a high-risk of ESKD have been also characterized with respect to DNA methylation. By analyzing DNA from about 300 patients with type 1 diabetes, Smyth et al. identified different methylation patterns associated with the phenotype of patients who progress to ESKD as compared with those who do not [74]. Genes involved in these patterns included *FKBP5*, *RUNX3*, *PIM1*, *ELMO1*, and *LY9*. Polymorphisms in these genes have been associated with cardiovascular and kidney disease, ageing, tumor cell proliferation, TGF-β signaling and inflammatory-immune pathways [74]. Type 2 diabetes is defined with the altered cellular response to insulin [75]. Type 2 diabetes is an extremely heterogeneous disease in terms of future prognosis. Traditionally, two kidney measures—eGFR and albuminuria—have been used in these patients to establish the future individual risk of developing kidney and CV outcomes [11]. However, it has also been demonstrated that, based on these two kidney measures only, a prediction of future prognosis is still imprecise [76]. Hence, from the perspective of precision medicine, clinical research has recently focused on finding novel biomarkers that improve risk prediction besides and beyond eGFR and albuminuria. In patients with type 2 diabetes, plasma levels of Tumor Necrosis Factor receptors (TNFR)-1 and TNFR-2 receptors are associated with an increased risk of CKD progression and ESKD in survival models adjusted for baseline eGFR and the urine albumin excretion rate, suggesting that this association may reveal a true (rather than merely statistical) pattern of disease. Thus, they may help to improve risk stratification of DKD patients [77]. Importantly, these two markers forecast ESKD even in the absence of proteinuria, thus testifying their possible predictive role in the earlier stages of CKD and in non-proteinuric phenotypes of CKD [78]. TNFR-1 and TNFR-2 activate pathways of inflammation and apoptosis and influence the levels of other inflammatory cytokines such as IL-1β and IL-6 [79]. It has been hypothesized that both TNF receptors have a direct toxic effect on the kidney. In fact, experimental and human studies in DKD have found that TNF-α mRNA expression was deeply associated with the development of glomerular and tubular lesions [77]. Subsequently, the TNFR patterns have been expanded with the discovery of a signature of 17 inflammatory proteins of the TNFR superfamily, namely the Kidney Risk Inflammatory Signature (KRIS), which accurately predicts the onset of ESKD irrespective of the severity of DKD and in heterogeneous populations [77]. Similar findings have been reported for the kidney injury molecule–1 (KIM-1). KIM-1 is a transmembrane protein expressed in the proximal tubular cells in the kidney and it has been shown to promote kidney fibrosis and to accelerate eGFR decline in patients with type 2 diabetes [80]. More importantly, it has been shown that the plasma KIM-1 level is associated with CKD progression strongly and independently of the TNFR-1 and -2 levels, in patients with both early and advanced DKD [81]. This pattern was indeed evaluated in the ACCORD cohort (early DKD) and in the VA-NEPHRON-D trial (advanced DKD), two large trials conducted in patients with type 2 diabetes and CKD [53,82]. The novel trials with SGLT2 inhibitors in DKD patients have helped the comprehension of the role of biomarkers in the underlying kidney damage. In a post-hoc analysis of the CANVAS trial, Sen et al. investigated the prognostic role of growth differentiation factor-15 (GDF-15) on kidney and CV outcomes [83]. In particular, they found that increased plasma levels of GDF-15 (the highest quartile or by doubling levels) were associated with a 20 to 30% higher risk for CV events, a 1.5 to 2.1 higher risk for the development of heart failure and up to a 3-fold higher risk for kidney outcome, respectively, in survival models adjusted for eGFR, albuminuria and many other confounders. In the same study, the treatment with canagliflozin reduced the mean level of GDF-15 as compared to baseline levels (start-of-treatment visit), yet the treatment effect on future outcomes did not depend on the GDF-15. GDF-15 is an inflammatory marker whose plasma levels increase in chronic conditions such as diabetes or CKD [84]. The prognostic effect of GDF-15 on CV and kidney events is mediated by several mechanisms, including endothelial NO synthase and NFĸB [85]. As for type 1 diabetes, several markers of CV risk are also used in patients with type 2 diabetes for predicting future CV and kidney outcomes [86]. These include high-sensitivity cardiac troponins (hs-cTnT and hs-cTnI) and (NT-proBNP) and are widely used in CKD patients to diagnose coronary artery disease and heart failure, respectively. In type 2 diabetes patients, the measurement of both hs-cTnT and hs-cTnI improves CV risk stratification [87]. Such evidence is of particular importance given the availability of cardioprotective drugs for these patients and also given the particular high CV risk due to sodium sensitivity, and the oxidative and inflammatory stress underlying DKD conditions [88]. Similarly, NT-proBNP, a biomarker of fluid retention, has been shown to predict CV and kidney endpoints in DKD patients [89]. High plasma copeptin levels were found to forecast CKD progression (ESKD or doubling of serum creatinine), in patients with type 2 diabetes [90]. Such an association was strong and independent of a series of baseline covariates such as age, gender, eGFR and albuminuria. The robust findings that copeptin impairs DKD prognosis both in type 1 and type 2 diabetes supports the pathogenetic role of vasopressin through the activation of a V2-receptor and perpetuates the implementation of novel promising drugs for these high-risk patients [91]. All these mentioned studies, carried out in type 2 diabetes patients, evaluated the additive prognostic and risk stratification role of single biomarkers, or biomarker families, on future cardiorenal endpoints. One further step forward was the assessment of more complex biomarkers, namely the combination of multiple markers that can be measured together and that are able to classify DKD patients according to their future risk, as high- and low-risk [20]. One important example in the context of DKD is represented by the CKD273. The CKD273 is a panel of 273 urine peptides that were originally developed to detect early the presence of CKD from any cause [92]. In DKD patients, the CKD273 was found to be able to predict the onset of albuminuria and CKD progression over time, thus correctly working as a classifier [93]. Owing to this finding, it has been used to carry out a randomized study, the proteomic prediction and renin angiotensin aldosterone system inhibition prevention of early diabetic nephropathy in type 2 diabetic patients in a normoalbuminuria (PRIORITY) trial, during which DKD patients were included in the study on the basis of their risk (assessed with the classifier) of developing albuminuria [94]. Only high-risk patients were randomized to receive spironolactone, an albuminuria-lowering drug, or placebo. This was an extremely interesting and leading study in the context of personalized medicine trials since, for the first time, an antialbuminuric treatment was given to patients not yet albuminuric but at an increased risk of developing it. Encouraging prognostic data in patients with type 2 diabetes also derive from genomic analysis. Genetic variants in the *UMOD* gene, encoding uromodilin, a protein synthetized in kidney tubules, was associated with CKD development in a multiethnic analysis [95]. From the same population, 13 variants predicted CV complications of type 2 diabetes patients. Polymorphisms in genes encoding cubilin and megalin modified ESKD risk in the African American population [96]. In a Chinese population of type 2 diabetes patients, 5 SNPs predicted eGFR decline over time, yet the association was weak [76]. One further interesting point in the precision nephrology perspective is that the vast majority of risk prediction models have been built thus far using standard regression techniques (e.g., Cox proportional hazard or other multivariable regression analyses) [20]. Such models furnish the adjusted risk associated with each covariate included in the model (for instance, an increment of 20% of risk of future events for each mg increase in ACR regardless of other risk factors) but do not provide individual risk and prognostic estimates. This is done, for example, with the risk scores, because they calculate the future risk for a patient considering the exact level of multiple covariates. For instance, a typical risk score can provide the 3-year risk (%) of ESKD of a 65 year-old patient, who is smoking 15 cigarettes per day, with 25 kg/m^2^ of body mass index, 290 mg/g of ACR and NT-proBNP of 423 pg/mL. Risk scores may facilitate the clinical decision making process for the clinicians but may also inform patients about their future risk. To be computed, a rigorous methodology is needed and the computation (and presentation as well) of individual risk prediction metrics such as discrimination, calibration, external validation and goodness-of-fit (GOF) must be measured. Very few studies have reported these measures in DKD, and generally in CKD, thus far [97]. In the past decade, Tangri and colleagues developed the Kidney Failure Risk Equation (KFRE), reporting that four variables—age, gender, eGFR, albuminuria—were, if taken together, sufficiently strong to predict ESKD risk [98]. Metrics of individual performance such as discrimination, GOF and calibration were reported for this equation and an electronic calculator was provided as well. Subsequently, we refined the KFRE by replacing the absolute proteinuria with proteinuria indexed to eGFR (proteinuria/eGFR*100, also called F-Uprot), which has been shown to predict more accurately ESKD risk in advanced CKD stages [26]. Further risk equations have been developed in patients with advanced stages of CKD only or in health-care insurance systems such as the Kaiser Permanente Northwest [99,100]. Specifically, in type 2 diabetes patients, a meta-analysis of 20 cohort studies was used to develop and validate a risk score for the onset of DKD [101]. This score highlighted the importance of traditional risk factors such as age, smoking habit and UACR in the risk prediction and all significant factors were then used to build a nomogram. Another risk score for the prediction of kidney function decline in DKD patients has been developed in the Mount Sinai Health System [102]. This score, called KidneyIntelX™ combined novel (KIM-1, TNFR-1 and 2) and traditional (serum calcium, albuminuria, eGFR) biomarkers and were demonstrated to be more accurate in terms of discrimination as compared to the KDIGO risk categories. Despite the demonstration and presentation of these scores, a further effort is needed to improve risk stratification and prognosis of DKD patients. An apparently minor, yet very important, point in this context is to spread individual risk prediction models within the nephrology community, encouraging the use of the available models to improve patients’ care [103].

## 6. Clinical and Genetic Predisposition to Individual Response to Therapies in Diabetes and CKD

**Figure 2:**
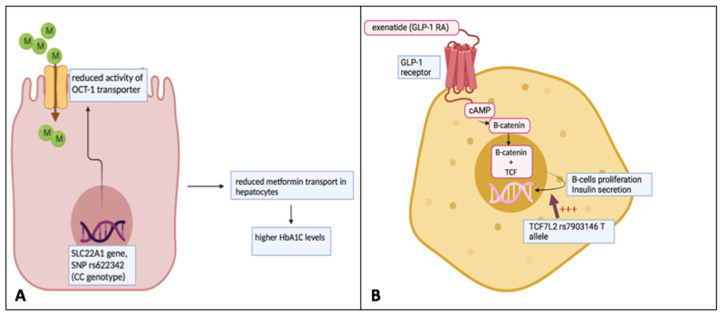
Individual variation of response to metformin and GLP-1 receptor agonists based on pharmacogenomic variants. (**A**) The variant rs622342 (CC) of the SLC22A1 gene leads to a decreased activity of the OCT-1 transporter across the cellular membrane. OCT-1 transporter is responsible for the intra-hepatic transport of metformin. The reduction in metformin amount into these cells may contribute to a minor response to the drug. (**B**) TCF7L2 is involved in the molecular pathway, which facilitates the GLP-1-dependent insulin secretion from pancreatic β-cells. Its genetic variant rs7903146 (T allele) is associated with a positive response to the GLP-1 receptor agonist exenatide. This figure was originally created by the authors.

The treatment of DKD is aimed at correcting multiple risk factors such as hyperglycemia, hypertension, albuminuria, lipid disorders and obesity. The long-term goal is to prevent the onset of CV complications and the kidney function progression to the more advanced and severe stages of the disease. Targeting these multiple risk factors is necessary to reach the goal. A crucial step in the ongoing clinical research into DKD is to understand which biological or clinical factors can predict the response to treatments [104]. Prognostic research can lead to the detection of subgroups of patients at high or very-high risk of future events, but it is detrimental to assess to what treatment these patient categories will respond in order to minimize their risk of long-term complications. Such a work setting perfectly fits with the aim of precision medicine in DKD patients. As depicted below, the available studies have reported a large individual variation in response to anti-diabetic treatments in type 2 diabetic patients. Metformin is considered the first-line treatment for hyperglycemia and in DKD patients, it is not contraindicated unless the kidney damage is advanced (i.e., eGFR < 30 mL/min/1.73 m^2^) or if conditions predisposing to lactic acidosis (heart failure, active liver disease, systemic hypoperfusion, sepsis) coexist. Clinical and pharmacogenetic factors explain the individual variation in response to metformin. It has been shown that older age, a lower body mass index (BMI) and the short duration of diabetes predict the positive response in terms of the magnitude of glycated haemoglobin (HbA1c) reduction to metformin [105,106]. This latter evidence supports the early initiation of metformin in diabetic patients. Genetic variants of the *SLC22A1* gene, encoding the organic cation transporter-1 (OCT-1) and the *SLC47A1* gene encoding multidrug and toxin extrusion 1 transporter (MATE-1), influence both pharmacokinetic (PK) and pharmacodynamic (PD) behavior of metformin. With respect to the *SLC22A1* gene, variant rs622342 (AA) has been shown to predict a greater glycemia-lowering response to metformin as compared to minor C variant in the same gene (Figure 2) [84,107]. This evidence can be explained by the lower OCT-1 activity in patients with C alleles. OCT-1 is a transporter located in the brush border of gut cells and in basolateral membranes of renal cells and hepatocytes and it is involved in important processes, such as metformin absorption from the intestine, and drug transport across hepatic and kidney cells. Regarding MATE-1, this is a transport located in the luminal membrane of proximal tubular cells in the kidney and in the biliary pole of the hepatocytes, where it mediates the efflux of metformin and other substrates. Hence, reduction in MATE-1 function, as in the case of the rs2289669 variant, predicted a greater response to metformin, which is likely associated with its increased plasma levels [107]. Similar studies have also demonstrated an individual variability in response versus the aforementioned nephroprotective treatments GLP1-RA and SGLT2 inhibitors. Polymorphisms in the GLP1 receptor gene exert different responses to GLP1-RA. The variant rs6923761 was associated with a greater response to liraglutide, whereas the polymorphism rs10305420 in the T allele has been associated with a lower response in terms of weight loss and Hb1Ac to the GLP1-RA exenatide [108,109]. Interestingly, polymorphisms in the *TCF7L2* gene were associated with a positive response to exenatide [110]. The *TCF7L2* gene is involved in the molecular pathway, which facilitates the GLP-1-dependent insulin secretion from the pancreatic β-cell [111,112]. Variants in this gene influence the response to GLP1-RA, but also to the dipeptidyl-peptidase-4 inhibitors [113].

The SGLT2 inhibitors are novel drugs which are certainly gaining momentum in the treatment of patients with diabetes and CKD. Some studies have highlighted a greater response in males than in females to these agents, even though this finding is still controversial [114]. One reason underlying this difference can be the higher expression of these transporters in males. Genetics play a relevant role in determining the degree of response to SGLT2 inhibitors as shown by the first studies examining these patterns. Variants in the *UGT1A9* gene such as UGT1A9*3 and UGT2B4*2 have been associated with a higher plasma concentration of canagliflozin and with a higher response to this drug [115]. Variants in the *SLC5A2* gene, encoding the SGLT2 transporter, have also been found but whether they determine a PD variation in drug response is still unclear [93,116]. Landmark trials have shown that SGLT2 inhibitor effect is consistent across many subgroups of patients [117]. However, about 20% of patients who started treatment with the SGLT2 inhibitor dapagliflozin did not respond, in terms of albuminuria reduction, in a previous trial, with this nonresponse being reproducible after re-exposure to the same drug [118]. Since the first real-life experiences of treatment with SGLT2 inhibitors are currently active, it would be extremely important to have in the next future studies showing who are the patients which do not sufficiently respond to the SGLT2 inhibitors. The reasons underlying the variability in response to ACEi and ARBs are several and encompass clinical and genetic determinants. Previous studies reported that patients with a high BMI and obesity have a decreased response to these agents, with endocrine and metabolic factors being involved in enhancing the low response [119,120]. Furthermore, the high sodium intake is associated with a low response to both ACEi and ARBs and this effect is also present with respect to the onset of future hard endpoints such as CV events and CKD progression over time [121]. Other than sodium, serum potassium also plays a pivotal role in response to ARBs. A post-hoc analysis of the RENAAL trial showed that patients who started the ARB losartan and developed hyperkalemia in the first few weeks were not more protected against the subsequent kidney risk (CKD progression to ESKD) as compared to those who did not develop hyperkalemia [122]. Genetic variants may also influence the response to ACEi and ARBs. An insertion (I) or deletion (D) polymorphism of the *ACE* gene modifies the activity of the systemic and renal renin-angiotensin-aldosterone system (RAAS) with a higher activity in patients with the D polymorphism. Response to ACEi and ARBs was found increased in patients with DD polymorphism, which also is the genotype associated with the highest risk of DKD progression [123,124]. Statins are widely used in patients with diabetes and CKD, since the presence of both conditions dramatically increases the risk of major CV events and CV death. These drugs work through the competitive inhibition of the enzyme 3-hydroxy-3-methylglutaryl-CoA reductase, responsible for the cholesterol biosynthesis in the liver. Statins lower LDL cholesterol levels, but a degree of individual variation in treatment effect has been found. Clinical and demographic variables such as older age, male gender and lower alcohol consumption have a larger LDL cholesterol reduction in response to statins. Conversely, smokers show a lower response to these drugs [125]. Polymorphisms in the gene involved in the PK of statins are major modifications of their individual response, particularly with respect to the cytochrome P450 expression. Patients with increased CYP3A4 will have a higher response to lovastatin, simvastatin or atorvastatin, whereas those with hyperexpression of CYP2C9 will likely respond more efficaciously to fluvastatin or rosuvastatin [126,127,128,129,130]. Table 1 summarizes the main findings derived from prognostic and predictive studies in patients with diabetes and CKD.

Intriguingly, all these studies have shown that patients have a different response to almost all the nephroprotective treatments used, this being true for the old and the more recent drug classes. At the same time, these studies answer the question of whether one or more factors modify the individual response to a defined treatment. It would be even more interesting, in future studies, to combine all this predictive information to reach out to a typical setting of individual features, which predict the response to a panel of treatments, with the aim of optimizing the control of the high number of risk factors of future risk in DKD patients.

## 7. Conclusions

Precision medicine is gaining momentum in the context of heterogeneous disease such as DKD. Moreover, this condition is associated with an extremely high risk of kidney and CV events and its global burden is marked by a continuous increase in prevalence. Precision medicine encompasses prognostic and predictive aspects in DKD. In fact, there is a great interest in searching biomarkers and individual risk prediction models which are able to improve the risk stratification of patients. Similarly, current research is making progress on the comprehension of the mechanisms that influence the individual response to nephro- and cardioprotective treatments. Finally, but still importantly, spreading communication of both observational and randomized study findings to the nephrology community is an urgent need since it may directly transfer the good result of clinical research in the potential good results of clinical management. Further studies around these disparate topics of the same matter are more than expected in the near future.

## Figures and Tables

**Table 1:** Prognostic and treatment response biomarkers in DKD patients.

*Type 1 Diabetes*			
Prognosis	Source	Biomarker/Variable	Findings and Interpretation
		*Single Biomarkers/Biomarkers family*	
	Tofte N et al. [64]	MR-proANP, NT-proBNP	They are associated with 2-fold increased risk of EKSD, CV events and all-cause mortality, regardless of the main traditional risk factors.
	Costacou T et al. [65]	hs-cTnT	Blood levels of hs-cTnT were associated (with about 40% more risk for each unit increase) with CV events over time.
	El Dayem SMA et al. [67]	Copeptin	Higher blood levels of copeptin are strictly associated with the development of atherosclerosis, arterial stiffness and kidney. damage. Patients with the highest levels of copeptin have concomitantly increased levels of albuminuria.
	Nakano D et al. [71]	Urinary AGT	Urinary levels of ATG predict eGFR decline and ESKD, regardless of baseline levels of albuminuria.
		*Genomic findings*	
	Salem RM et al. [73]	Single nucleotide polymorphisms- 16 loci (e.g., SNP variant rs55703767)	SNP variant rs55703767 is responsible for a mutation in the collagen type IV alpha 3 chain (COL4A3). It was the variant with the strongest association with kidney damage and CKD progression.
	Smyth LJ et al. [74]	DNA methylation patterns *FKBP5-RUNX3-PIM1-ELMO1-LY0*)	Polymorphisms in these genes have been associated with cardiovascular and kidney disease, ageing, tumor cell proliferation, TGF-β signaling and inflammatory-immune pathways.
***Type 2 Diabetes***			
**Prognosis**	**Source**	**Biomarker/Variable**	**Findings and Interpretation**
		*Single Biomarkers/Biomarkers family*	
	Niewczas MA et al. [77] Waijer SW et al. [78]	TNFR-1/TNFR-2	Their plasma levels are associated with an increased risk of CKD progression and ESKD. They may help to improve risk stratification of DKD patients and forecast ESKD even in the absence of proteinuria, thus testifying their possible predictive role in the earlier stages of CKD and in non-proteinuric phenotypes of CKD.
	Nowak N et al. [80] Coca SG et al. [81]	KIM-1	Promote kidney fibrosis and accelerate eGFR decline. Plasma KIM-1 level is associated with CKD progression strongly and independently of the TNFR-1 and -2 levels and both in patients with early and advanced DKD.
	Luan HH et al. [84] Sen T et al. [83]	GDF-15	GDF-15 increases in chronic conditions such as diabetes or CKD. Increased plasma levels are associated with higher risk for CV events.
	Tang O et al. [87]	hs-cTnT/hs-cTnI	In DKD patients, the measurements of both biomarkers improve CV risk stratification.
	Kammer M et al. [89]	NT-proBNP	Predict CV and kidney endpoints.
	Velho G et al. [90]	Copeptin	High plasma levels were found to forecast the CKD progression (ESKD or doubling of serum creatinine). Such an association was strong and independent of a series of baseline covariates such as age, gender, eGFR and albuminuria.
		*Combination of multiple markers*	
	Roscioni et al. [93]	CKD273	Panel of 273 urine peptides that predict the onset of albuminuria and CKD progression over time.
		*Genomic findings*	
	Vujkovic M et al. [95]	*UMOD* gene	Genetic variants in *UMOD* gene were associated with CKD development in a multiethnic analysis. From the same population, 13 variants predicted CV complications of type 2 diabetes patients
	Ma J et al. [96]	Cubilin and Megalin genes	Polymorphisms in these genes modified ESKD risk in an African American population.
***Treatment Response Markers***			
	Nichols G.A. et al. [105] Becker M.L. et al. [107]	Metformin	First line treatment for hyperglycaemia. In DKD patients were not contraindicated unless the kidney damage is advanced or conditions predisposing to lactic acidosis coexist. Clinical and pharmacogenetic factors explain the individual variation of the response to metformin. Genetic variants of the *SLC22A1* and *SLC47A1* gene influence both pharmacokinetic (PK) and pharmacodynamic (PD) behavior of metformin.
	De Luis D.A. et al. [108] Ferreira M.C. et al. [110] Shu L. et al. [111]	GLP1-RA	Polymorphisms in the GLP1 receptor gene exert a different response to GLP1-RA.
	Nagai K. Et al. [114] Hoeben E. et al. [115] Zimdahl H. et al. [116]	SGLT2 inhibitors	Novel drugs in the treatment of patients with diabetes and CKD. Some studies have highlighted a greater response in males than in females. Genetic plays a relevant role in determining the degree of response to SGLT2 inhibitors.
	Cohen J.B. at al. [119] Kwaker Naak A.J et al. [121] Miao Y. et al. [122] Parving H.H. et al. [123]	ACE/ARB	Clinical and genetic reasons explain the variability in response to ACEi and ARBs. BMI and obesity, for example, are associated with a decreased response to these agents. An insertion (I) or deletion (D) polymorphism of the ACE gene modifies the activity of the systemic and renal renin-angiotensin-aldosterone system (RAAS) with a higher activity in patients with the D polymorphism.
	Simon J.A. et al. [125] Elens L. et al. [126]	Statins	Statins work through the competitive inhibition of the enzyme 3-hydroxy-3-methylglutaryl-CoA reductase, lowering LDL cholesterol levels. A degree of individual variation in treatment effect has been found. Polymorphisms in the gene involved in the PK of statins are majorly modificatory of their individual response, particularly with respect to the cytochrome P450 expression.

## Data Availability

Not applicable.
